# The Plant Defensin Ppdef1 Is a Novel Topical Treatment for Onychomycosis

**DOI:** 10.3390/jof9111111

**Published:** 2023-11-17

**Authors:** Nicole L. van der Weerden, Kathy Parisi, James A. McKenna, Brigitte M. Hayes, Peta J. Harvey, Pedro Quimbar, Sean R. Wevrett, Prem K. Veneer, Owen McCorkelle, Shaily Vasa, Rosemary Guarino, Simon Poon, Yolanda M. Gaspar, Michael J. Baker, David J. Craik, Rob B. Turner, Marc B. Brown, Mark R. Bleackley, Marilyn A. Anderson

**Affiliations:** 1 La Trobe Institute for Molecular Science, La Trobe University, Melbourne, VIC 3086, Australia; 2 Hexima Ltd., La Trobe University, Melbourne, VIC 3086, Australia; 3 Institute for Molecular Bioscience, The Australian Research Council Centre of Excellence for Innovations in Peptide and Protein Science, The University of Queensland, Brisbane, QLD 4072, Australia; 4 MedPharm Ltd., Surrey Research Park, Surrey GU2 7AB, UK

**Keywords:** plant defensin, onychomycosis, *Trichophyton rubrum*, antifungal, NMR, Ppdef1

## Abstract

Onychomycosis, or fungal nail infection, causes not only pain and discomfort but can also have psychological and social consequences for the patient. Treatment of onychomycosis is complicated by the location of the infection under the nail plate, meaning that antifungal molecules must either penetrate the nail or be applied systemically. Currently, available treatments are limited by their poor nail penetration for topical products or their potential toxicity for systemic products. Plant defensins with potent antifungal activity have the potential to be safe and effective treatments for fungal infections in humans. The cystine-stabilized structure of plant defensins makes them stable to the extremes of pH and temperature as well as digestion by proteases. Here, we describe a novel plant defensin, Ppdef1, as a peptide for the treatment of fungal nail infections. Ppdef1 has potent, fungicidal activity against a range of human fungal pathogens, including *Candida* spp., *Cryptococcus* spp., dermatophytes, and non-dermatophytic moulds. In particular, Ppdef1 has excellent activity against dermatophytes that infect skin and nails, including the major etiological agent of onychomycosis *Trichophyton rubrum*. Ppdef1 also penetrates human nails rapidly and efficiently, making it an excellent candidate for a novel topical treatment of onychomycosis.

## 1. Introduction

Onychomycosis is a persistent fungal infection of the nail, distinguished by nail discolouration, thickening, and distortion, and affects about 5–10% of the population globally [1,2,3]. These infections are hard to treat, can be painful, and have a cosmetic impact on the patient. Current topical treatments for onychomycosis require daily or weekly application for 48 weeks, and even these exhaustive treatment regimens produce low complete cure rates. Until 2014, Penlac^®^ and its generic equivalents, with the active ingredient ciclopirox, were the only prescription strength topical onychomycosis treatments approved by the FDA. The complete cure rate for Penlac^®^ is 5–9% [4]. Two new active ingredients for topical products were launched in 2014. Jublia^®^, with the active ingredient efinaconazole, has a complete cure rate of 15–18% [5]. Kerydin, with the active ingredient tavaborole, has a complete cure rate of 6–9% [6]. Several less effective over-the-counter treatments are also available, but there remains a need for more effective drugs for the treatment of onychomycosis. The low cure rate for current topical treatments of onychomycosis is associated with their hydrophobic properties and associated poor penetration through the hydrophilic matrix of the nail [7,8,9]. Consequently, relatively low levels of the active components reach the underside of the nail where the fungal infection is established in the nail bed.

Several systemic (oral) treatments have been approved for the treatment of onychomycosis. In the US, they include itraconazole (Sporanox^®^) and terbinafine (Lamisil^®^) with cure rates of 14% and 38%, respectively, after a 12 week treatment [10]. The FDA reported in its review of efinaconazole (Center for Drug Evaluation and Research; Application Number: 203567Orig1s000) that oral itraconazole or terbinafine are generally used to treat onychomycosis, but drug–drug interactions limit their use in some patients, especially the elderly who frequently have concomitant medications. Other limitations include safety concerns such as liver toxicity and the associated potential need for laboratory monitoring. 

A feature of the onychomycosis market is the low proportion of sufferers that access treatment. In the USA, in 2012, an estimated 35 million people suffered from onychomycosis (www.jubliarx.com/toenail-fungus, URL accessed on 16 October 2023), but it is often overlooked or undertreated [11]. This behaviour is driven by the high cost and low success rates of current treatments, long treatment times (48 weeks for topical products), a lack of awareness about the condition, and the undesirable side-effects of oral treatments. As such, there are distinct advantages for pursuing the development of effective topical products for the treatment of the disease.

Plant defensins are a large family of small, cationic, cystine-rich proteins that are part of the defence arsenal used by plants for protection against potentially damaging fungal infections [12,13,14]. Plant defensins represent a novel class of therapeutic molecules with several attributes that make them suitable for use as drugs [15]. Although none have yet been approved as drugs, their cystine-rich structure renders them stable to extremes of pH and temperature [16,17]. Plant defensins are also hydrophilic, making them relatively simple to formulate without the need for complex and toxic solvent systems [18]. Here, we describe the identification of a novel plant defensin, Ppdef1, suitable for the topical treatment of onychomycosis. Ppdef1 is from the plant *Picramnia pentandra*, otherwise known as Florida bitter bush, and has potent antifungal activity against a range of human fungal pathogens, including dermatophytes that infect skin and nails. Ppdef1 was selected over other plant defensins based on its low off-target activity on mammalian cells and excellent yield from recombinant expression [19]. We examined the antifungal activity of Ppdef1 in an infected nail model and nail fragments to assess the capability of Ppdef1 to penetrate human nails and inhibit fungi under the nail plate. We also assessed whether Ppdef1 maintains its activity in the presence of keratin and could penetrate human nails more efficiently than other antifungal drugs. The mechanism of action of Ppdef1 and its activity relative to other plant defensins is described in Parisi et al., 2023 [20]. 

## 2. Materials and Methods

### 2.1. Sequence Alignment

The Ppdef1 sequence was used to query the NCBI protein database using BLASTp. Several known plant defensin sequences, including one identified through the BLASTp search, were used to construct a CLUSTALW alignment using the program MUSCLE-Multiple sequence alignment. The percent sequence identity between proteins was also calculated using MUSCLE. 

### 2.2. Strains and Culture Conditions

*Trichophyton rubrum* and *Trichophyton mentagrophytes* were clinical isolates obtained from the National Mycology Reference Centre, South Australia Pathology, Australia. The *Candida albicans* strain used was ATCC 90028. *Trichophyton* spores were germinated onto half-strength Sabouraud dextrose agar (1/2 SDA) and grown for at least two weeks at 28 °C (with UV light) to generate spores prior to use. *C. albicans* was stored as a glycerol stock and streaked onto 1% yeast extract, 2% peptone, 2% dextrose (YPD) agar plates. Plates were incubated overnight at 30 °C and then used as the source for overnight cultures (in YPD, 30 °C, 250 rpm). *Fusarium oxysporum* f. sp. *vasinfectum,* Australian isolate VCG01111 isolated from cotton (gift from Wayne O’Neill, Farming Systems Institute, Department of Primary Industries (DPI), Queensland, Australia), was maintained on half-strength potato dextrose agar (1/2 PDA) plates at 26 °C to generate spores. 

### 2.3. Recombinant Expression of Ppdef1 Using Pichia pastoris

Ppdef1 was produced using the methylotrophic yeast *Pichia pastoris*. A DNA fragment encoding the mature Ppdef1 sequence along with an XhoI restriction endonuclease site, a KEX2 cleavage site at the 5′ end, a NotI restriction endonuclease site, and a stop codon at the 3′ end was purchased from Genscript for cloning into the pPIC9 vector. The STE13 protease site between the KEX2 cleavage site and the mature protein was removed to prevent the addition of Glu-Ala repeats at the N-terminus of Ppdef1. An alanine was added to the N-terminus to ensure an efficient cleavage by KEX2. DNA was then inserted into the pPIC9 plasmid via the XhoI and NotI restriction sites using standard protocols. The pPIC9 plasmid containing the gene of interest was linearized using the SalI restriction enzyme. Electrocompetent *P. pastoris* GS115 cells (Invitrogen, Waltham, MA, USA) were prepared as described by Chang et al. [21], and linearized DNA was transformed into these cells using standard protocols. Ppdef1 was then expressed in a BioFlo/CelliGen 115 fermentor/bioreactor (New Brunswick, Eppendorf, Melbourne, Victoria, Australia) and a 3L vessel via constant feed methanol induction in basal salts media according to the manufacturer’s instructions. Cells were removed from the fermentation medium with centrifugation at 2826× *g* in an Avanti J-E centrifuge (Beckman Coulter, Mt Waverley, Victoria, Australia), and the Ppdef1 protein was purified from the supernatant via cation-exchange chromatography using Fast-Flow SP-sepharose (Cytiva, Mt Waverley, Victoria, Australia) as described by Lay et al. [22] and size exclusion chromatography using HiLoad 26/600 Superdex 30 prep grade (GE). The protein concentration was determined using the bicinchoninic acid (BCA) protein assay (Pierce). Protein quality and purity was assessed using SDS-PAGE, MALDI-TOF MS, and RP-HPLC.

### 2.4. NMR

Ppdef1 was dissolved in 10% D_2_O/H_2_O at a concentration of ~1 mM and a pH of 3.5. NMR spectra were recorded at 298K on a Bruker Avance III 600 spectrometer (Bruker, Preston, Victoria, Australia). Chemical shifts of backbone and sidechain resonances were assigned by analysis of 2D TOCSY (with an 80 ms MLEV-17 spin lock), NOESY (mixing time of 200 ms), ECOSY, and natural abundance ^13^C and ^15^N HSQC experiments. Solvent suppression was achieved using excitation sculpting. Slowly exchanging amides were identified with slow D_2_O exchange and the sensitivity of amide chemical shifts to temperature. Spectra were processed using Topspin 3.5 and then analysed using CcpNmr Analysis. Chemical shifts were referenced to internal DSS (4,4-dimethyl-4-silapentane-1-sulfonic acid). 

### 2.5. Structural Analysis

Distance restraints were derived from NOESY spectra recorded at 289 K in both 10% and 100% D_2_O and used to generate initial structures with CYANA. Additional restraints included disulfide bonds; hydrogen bonds as indicated by preliminary structures and slowly exchanging amides; χ1 restraints from ECOSY and NOESY data; and backbone φ and ψ dihedral angles predicted by the program TALOS-N. CNS was then used to generate a final set of structures using torsion angle dynamics and refinement and energy minimization in explicit solvent. Final structures were assessed for stereochemical quality using MolProbity. The solvent accessible surface charge of Ppdef1 was mapped using the PDB2PQR v2.1.1 software [23] and the APBS v1.5 software [24]. Structural figures were generated using PyMOL v2.1.1 [25]. 

### 2.6. Antifungal Activity Assays

The minimum inhibitory concentration (MIC) and minimum fungicidal concentration (MFC) were determined using the broth dilution method described by Broekaert et al. [26]. MFC calculations were also adapted from the method described by Morace et al. [27]. All assays were repeated at least 3 times. Briefly, *T. rubrum*, *T. mentagrophytes*, or *F. oxysporum* spores were collected, filtered, counted, and adjusted to 5 × 10^4^ spores/mL with sterile half-strength potato dextrose broth (1/2 PDB). A two-fold serial dilution of Ppdef1 from 30 µg/mL was added to the wells in triplicate (20 µL/well). Half-strength PDB (20 µL) was added to control wells. Fungal spores were then added to the wells (80 µL of 5 × 10^4^ cells/mL, total 4000 cells). The optical density at 595 nm (OD_595_) was measured as the t = 0 value. Plates were incubated at 30 °C prior to another OD_595_ reading at 72 h. Growth was monitored by subtracting the OD_595_ reading at t = 0 from OD_595_ at t = 72 h and compared to the no-protein control. MIC was determined as the lowest concentration at which no growth was observed by eye. 

For the *C. albicans* experiments, YPD broth was inoculated with *C. albicans* prior to incubation overnight at 30 °C with shaking at 300 rpm. The number of cells were counted using a haemocytometer and diluted to 5000 cells/mL with ½ PDB. A two-fold serial dilution of Ppdef1 from 30 µg/mL was added to the wells in triplicate (20 µL/well). Half-strength PDB (20 µL) was added to control wells. The *C. albicans* preparation (80 µL; 5000 cells/mL, total 400 cells) was then added to each well of the plate containing the antifungal drug or no protein controls. The OD_595_ was measured at t = 0, and after 20 h, the plate was incubated at 30 °C. Growth was monitored by subtracting the OD_595_ reading at t = 0 from OD_595_ at t = 20 h and compared to the no-protein control. IC_50_ was calculated as the concentration of protein that inhibited 50% of growth, and the MIC was determined as the lowest concentration at which no growth was observed by eye.

From the MIC experiment, MFC values were calculated by assessing the wells where there was no visible growth for viable colony forming units after spreading the contents of the wells on agar plates. For each well where there was no visible growth, 80 µL was collected and transferred into sterile 1.5 mL tubes. Cells were pelleted using centrifugation, and the supernatant was removed before the cells were resuspended in 80 µL of sterile phosphate-buffered saline (PBS). The entire sample was then plated onto ½ SDA plates (*T. rubrum or T. mentagrophytes*) or YPD agar plates (*C. albicans*) and incubated at 30 °C for 18 h (*C. albicans*) or five days (*T. rubrum* or *T. mentagrophytes*) before plates were assessed for growth of fungal colonies. Plates with fewer than three colonies were recorded as ‘no growth’.

### 2.7. Propidium Iodide Uptake in Response to Antifungal Treatment

A culture of *C. albicans* cells (OD_600_ = 0.3 in ½ PDB) was prepared. Aliquots of the culture (300 µL) were transferred to 1.5 mL tubes and treated with 3.125, 6.25, 12.5, 25, 50, or 100 µg/mL of Ppdef1, efinaconazole, ciclopirox, terbinafine, or tavaborole. Water or dimethyl sulfoxide (DMSO) was added to the untreated control samples. Propidium iodide (2 µL of 1 mg/mL stock) was also added to each sample, and the cells were incubated for 30 min at 30 °C before they were pelleted using centrifugation, resuspended in 300 µL of PBS and transferred to the wells of a 96-well plate. Cells were assessed for fluorescence using the PI channel on a FACS Canto 2 flow cytometer (BD). Cells with fluorescence of more than 185 rfu were considered positive for PI uptake.

### 2.8. Antifungal Activity in the Presence of Keratin

Growth inhibition assays with *T. rubrum* were conducted as described above in the absence or presence of ground nail material (2 mg/mL) or hydrolysed keratin (2 mg/mL). To produce ground nail material, human nail clippings were cut into fragments of <3 mm squares and ground using a Bio-Gen PRO200 tissue homogenizer (PRO Scientific, Oxford, USA), until a fine powder was achieved. Hydrolysed animal keratin from animal skin was purchased from CNLabs (China). Keratin was added to the assay medium and then sterilized at 99 °C and cooled to room temperature prior to use in the assay. 

### 2.9. Cell Killing of T. rubrum Growing on Nail Fragments

Human nail fragments (~2 mm × 2 mm) were sterilized in water at 99 °C for 4 h. A single nail fragment was placed into each well of a 96-well microtiter plate along with 100 µL of sterile water. *T. rubrum* conidia (100 µL) were added to obtain a final concentration of 1.5 × 10^5^ conidia/mL. Control wells were inoculated with water only. Plates were then incubated for 96 h at 28 °C without shaking to allow *T. rubrum* to colonise the nail material. After the nail fragments were colonised with *T. rubrum*, they were treated with Ppdef1 or efinaconazole at a final concentration of 100 or 200 µg/mL. Six replicate wells were used per treatment, and two independent replicates were performed. As efinaconazole was dissolved in ethanol, all wells treated with Ppdef1 were adjusted with ethanol to a final concentration of 1% ethanol. Plates were then incubated for 20 h at 28 °C. 

After the 20 h incubation, 20 µL of PrestoBlue^®^ (Thermo Fisher, Scoresby, Victoria, Australia) was added to each well, and the mixture was transferred to a black-wall, clear-bottom microplate. PrestoBlue^®^ is converted to a fluorescent product by metabolically active cells and is therefore a measure of cell viability. The reaction was allowed to proceed for 24 h before fluorescence was measured in an M5e spectrophotometer (Molecular Devices, San Jose, CA, USA) using an excitation wavelength of 560 nm and an emission wavelength of 590 nm as described in the manufacturer’s instructions (Thermo Fisher, Scoresby, Victoria, Australia). 

### 2.10. Nail Penetration Assays

Nail penetration assays were conducted essentially as described by Hui et al. [28]. Human fingernail clippings (minimum size: 6.5 mm × 6.5 mm) were hydrated by placing them on folded Kimwipe tissues soaked in 3 mL of phosphate-buffered saline (PBS, pH 7.4) for 3 h at 26 °C. After hydration, nails were placed in plastic Franz cell nail adaptors (Perme Gear, Hellerton, PA, USA) with cotton wool (20 mg ± 2 mg) pre-soaked with 100 µL of phosphate-buffered saline with Tween 0.05% (PBST, pH 7.4) placed underneath the nail. Nails were sealed into the adaptors with silicone and checked for leaks by applying 10 µL of water to the top of the nail for at least 20 min (RT). Ppdef1 drug product (10 µL; 1% Ppdef1 in 50 mM acetate buffer, pH 4.0, 20% isopropanol, 20% propylene glycol), terbinafine (1% in 50 mM acetate buffer, pH 4.0, 20% isopropanol, 20% propylene glycol), or efinaconazole (formulated as Jublia (Valeant)) was applied to the top of the nail, and nails were incubated at 26 °C for 16 h in a Petri dish. A moist Kimwipe was added to the Petri dish to maintain humidity. After 16 h, the cotton wool was removed from the underside of the nail and set aside for analysis. The top of the nail was washed to collect excess test compound that had not penetrated the nail. First, a dry cotton swab was used to collect any excess liquid on the nail. A second swab soaked in 100 µL of water was then wiped over the top of the nail. An additional dry swab was used to collect any remaining liquid. A fresh piece of cotton wool soaked in 100 µL PBST was added to the underside of the nail. The nail was incubated at 26 °C for 1 h to allow for rehydration. Additional test solution (10 µL) was applied, and the process was repeated daily for 10 days. To quantify the amount of test item that had penetrated the nail, cotton wool removed from the underside of the nail was washed twice with 500 mM CaCl_2_ (200 µL) and then once with 0.1% TFA (200 µL). The cotton wool was incubated with the wash solution for 10 min at 26 °C for each wash. Cotton wool washes were pooled and analysed using RP-HPLC (300SB-C8 analytical column attached to a 1200 series HPLC (Agilent, Santa Clara, CA, USA)). Ethics approval for use of the nail clippings was granted by the La Trobe University Human Ethics Committee for the collection of nail clippings (approval number: HEC18414). 

### 2.11. Infected Nail Model

The infected nail model was conducted by MedPharm (Surrey, UK) as described in [29]. The test items used are as follows: Ppdef1 reduced drug product (1% Ppdef1 in 50 mM acetate buffer, pH 4.0, 0.2% glycerol, 20% propylene glycol), Jublia^®^ (10% efinaconaole solution), Penlac^®^ (Ciclopirox 8% topical solution), and Ppdef1 vehicle (50 mM acetate buffer, pH 4.0, 0.2% glycerol, 20% propylene glycol). Ten microlitres of each test item was added daily for seven days to the nails infected with a clinical isolate of *T. rubrum*. After seven days treatment, the relative level of viable fungi remaining was assessed by monitoring ATP levels using a previously validated bioluminescence method [30].

### 2.12. Statistical Analysis

Statistical analysis was performed using Graph Pad Prism version 10.0.2. Standard T-tests were used to assess statical significance, and the results relevant to the discussion are reported in the figure legends.

## 3. Results

### 3.1. Ppdef1 Is a Member of the Plant Defenisn Family

**Figure 1:**
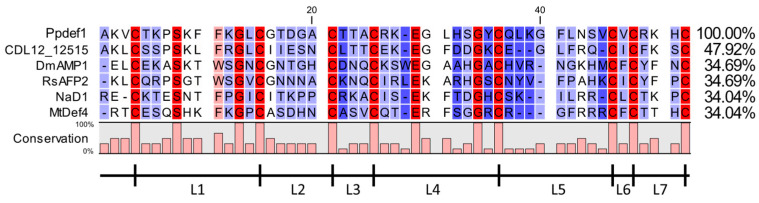
Sequence alignment of Ppdef1 with several members of the plant defensin family. MUSCLE alignment of known plant defensins CDL12_12515 (from *Handroanthus impetiginosus* PIN14865.1), DmAMP1 (from *Dahlia merckii* P0C8Y4.1), RsAFP2 (from *Raphanus sativus* AAA69540.1), NaD1 (from *Nicotiana alata* Q8GTM0.1), and MtDef4 (from *Medicago truncatula* XP_003628977.1) with Ppdef1. Amino acid identity and similarity are indicated by coloured shading. Conserved residues are coloured red. Gaps have been inserted to maximize alignment. Loops are defined as regions between cysteine residues (L1–L7).

Ppdef1 is a novel protein from the Florida bitter bush, *Picramnia pentandra* (Picramniaceae). A BLASTp search against the NCBI non-redundant database revealed that it was a member of the plant defensin family, and its closest other member, CDL12_12515 (from *Handroanthus impetiginosus* PIN14865.1), has a 48% identity after optimal alignment (Figure 1). The other defensins in the alignment share 34% sequence identity to Ppdef1, and all have the eight conserved cysteines that define the defensin family (Figure 1). The loop 5 region of Ppdef1, between β-strands 2 and 3, is 9 amino acids in length, which is longer than most defensins that typically have a loop 5 sequence of 5–8 amino acids.

### 3.2. Ppdef1 Forms the Conserved Cysteine Stabilised αβ Motif Typical of Plant Defensins

**Figure 2:**
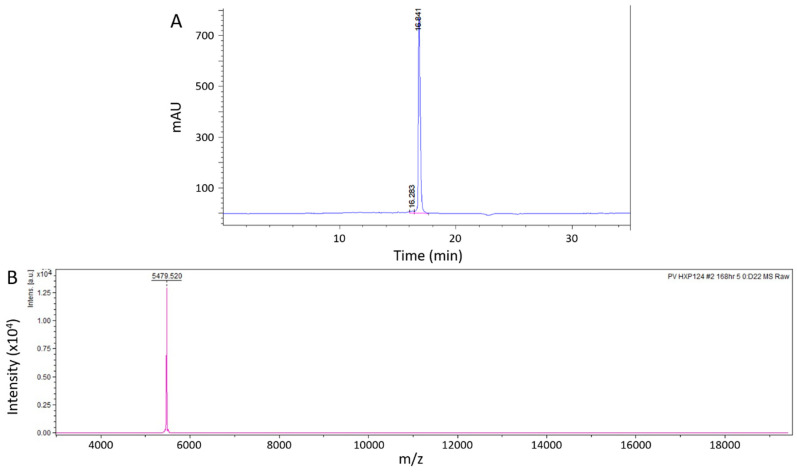
Quality assessment of recombinant Ppdef1. Ppdef1 was expressed in *P. pastoris* and purified using cation exchange chromatography followed by size exclusion chromatography. (**A**) The final purified material yielded a single peak when analysed with RP-HPLC. (**B**) MALDI-TOF analysis confirmed that the purified material contained a single species with a mass that is consistent with the expected mass of the folded defensin (5478.5 Da).

The genetic sequence encoding Ppdef1 was recombinantly expressed using the methanol-inducible pPIC9 system in *P. pastoris* using a 3 L fermentor. Defensin was purified from the fermentation supernatant using cation exchange chromatography followed by size exclusion chromatography. The final product was confirmed to have the expected mass and to be highly pure using MALDI-TOF-MS and RP-HPLC (Figure 2). 

**Figure 3:**
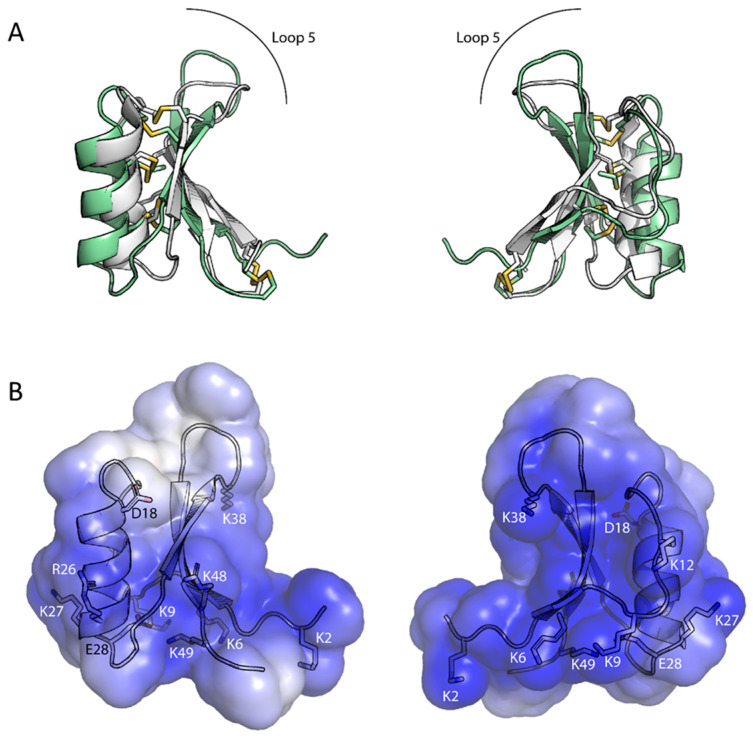
NMR solution structure of Ppdef1. (**A**) Cartoon representation of Ppdef1 (green) overlaid with NaD1 (grey, PDB ID: 1MR4), showing the extended loop 5 of Ppdef1. (**B**) Solvent accessible surface of Ppdef1 coloured according to its electrostatic potential at pH 7.0 (scale from −5 kT/e (red) to +5 kT/e (blue)). Charged residues are numbered with sidechains represented as sticks.

The three-dimensional solution structure of Ppdef1 was determined using NMR spectroscopy. The overall fold of Ppdef1 is consistent with the defensin defining cysteine stabilized αβ motif [31] (Figure 3). NMR spectra showed a good amide dispersion, and backbone resonances were fully assigned apart from the two residues at the N-terminus, Ala1 and Lys2, as well as Lys38. The structure of Ppdef1 was calculated with a total of 419 distance restraints, 82 dihedral angle restraints, and 24 hydrogen bond restraints. Amide temperature coefficients and deuterium exchange experiments were used to identify residues taking part in hydrogen-bond interactions (C21, T23, A24, C25, R26, K27, G29, Y34, V44, V46, C47, and R48), further supporting the identified secondary structural elements. The following disulfide connectivities were also included as restraints in the structure calculations: Cys4-Cys51; Cys15-Cys35; Cys21-Cys45; and Cys25-Cys47. The single proline residue (P7) adopted the trans conformation, as evidenced by strong H^δ^(*i*)Pro-H^α^(*i* − 1) signals in NOESY spectra and the ^13^C shifts of the C^β^ and C^γ^ proline resonances. The resulting family of structures overlaid well, particularly when fitted across the residues involved in the helix and β-strands, with a RMSD for the backbone atoms of 0.61 Å. Analysis of these structures revealed that 95% of the residues fell in the most favoured regions of the Ramachandran plot and a mean overall MolProbity score of 2.1 indicated a reasonable structural quality (Table S1). 

Ppdef1 adopts a typical cysteine-stabilized αβ motif with an α-helix spanning nine residues from Asp18—Arg26 and a triple-stranded anti-parallel β-sheet (β1 = Thr5—Pro7; β2 = Ser32—Leu37; β3 = Ser43—Arg48). There is some flexibility in the N-terminus as well as the beta hairpin turn formed by the loop between the β2 and β3 strands. An overlay of the solution structure of Ppdef1 with that of the plant defensin NaD1 (PDB-1MR4) demonstrates that the structure of Ppdef1 is highly similar to that of other plant defensins apart from an extension of the β2 and β3 strands and the hairpin loop that joins them (Figure 3A). Ppdef1 has a net positive charge of +7 at neutral pH. Analysis of the charge distribution across the surface of the protein revealed that the molecule bears a mostly positive charge, except for a small neutral patch on one side of the protruding loop 5 (Figure 3B). 

### 3.3. Ppdef1 Is Active against a Broad Range of Human Fungal Pathogens That Cause Fungal Nail Infections

Ppdef1 was tested for growth inhibitory and fungicidal activity against a range of human fungal pathogens that cause fungal nail infections including *T. rubrum*, *T. mentagrophytes*, *C. albicans*, and *F. oxysporum* (Table 1). Ppdef1 inhibited the growth of all pathogens tested with a minimal inhibitory concentration (MIC) between 13 and 30 µg/mL. To determine whether Ppdef1 has a fungistatic or fungicidal mode of action, the concentration at which no viable fungal cells remained (minimal fungicidal concentration, MFC) was also determined. In all cases, the MFC for Ppdef1 was the same or only marginally higher than the MIC (Table 1). 

### 3.4. Ppdef1 Kills C. albicans Cells within 30 min

**Figure 4:**
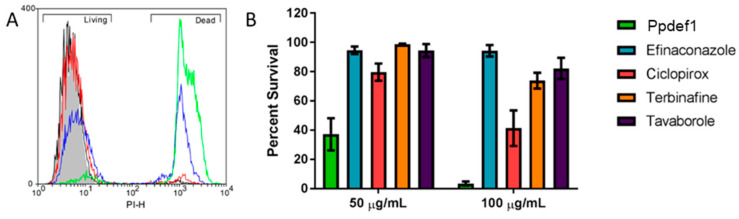
Ppdef1 permeabilizes *C. albicans* cells within 30 min. *C. albicans* cells were treated for 30 min with Ppdef1 and then assessed for membrane permeabilization using PI. (**A**) Flow cytometry analysis of cells treated with 0 (grey filled), 12.5 (black line), 25 (red line), 50 (blue line), or 100 µg/mL (green line) Ppdef1. A concentration-dependent increase in the proportion of dead cells (right peak) was observed. (**B**) Percent survival (corresponding to the left peak in (**A**)) of *C. albicans* cells treated with either 50 or 100 µg/mL of various antifungal drugs.

Fungal cell killing by Ppdef1 was compared to other antifungal drugs by measuring the uptake of the membrane impermeable dye, Propidium Iodide (PI). Ppdef1 induced the uptake of PI into *C. albicans* cells in a dose-dependent manner (Figure 4). After a 30 min treatment with 50 µg/mL Ppdef1, an average of 62.9% of the treated cells were no longer viable. At 100 µg/mL Ppdef1, this figure increased to 96.5%. In contrast, the other antifungal drugs were less efficient at killing *C. albicans* cells. At 100 µg/mL, ciclopirox killed only 58.7% of cells and terbinafine and tavaborole killed less than 25% of cells. Efinaconazole did not induce a significant cell death at any of the tested concentrations.

### 3.5. Ppdef1 Retains Activity against T. rubrum in the Presence of Keratin

**Figure 5:**
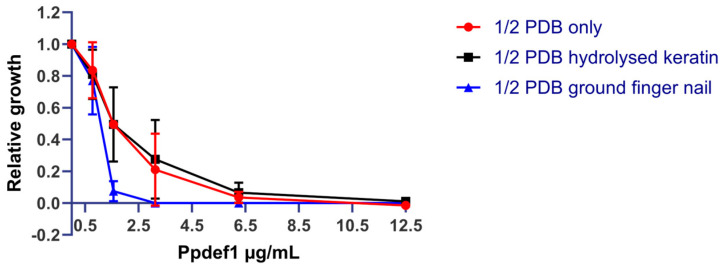
Ppdef1 retains activity in the presence of keratin. Growth of *T. rubrum* hyphae was monitored after treatment with increasing concentrations of Ppdef1 in either ½ PDB (red), ½ PDB with 2 mg/mL hydrolysed keratin (black), or ½ PDB with 2 mg/mL ground human fingernail (blue). Ppdef1 decreased the growth of *T. rubrum* in a concentration-dependent manner in all the growth conditions assessed. Relative growth was calculated relative to the untreated fungi in each medium. Error bars represent the standard error for the mean of the two biological replicates.

To evaluate whether Ppdef1 retains activity in the clinically relevant setting of the nail bed, *T. rubrum* in ½ PDB was challenged with Ppdef1 in the absence or presence of either hydrolysed keratin or ground nail fragments. After 92 h, the viability of the fungus was assessed using PrestoBlue^®^. *T. rubrum* grew at least 2-fold faster in medium containing hydrolysed keratin or ground nail than it did in ½ PDB alone. The Ppdef1 MIC in ground nail (3.125 µg/mL) was approximately 4-fold lower than the MIC in either ½ PDB alone (12.5 µg/mL) or ½ PDB with hydrolysed keratin (12.5 µg/mL) (Figure 5), indicating that Ppdef1 not only retains activity but is more active in the presence of keratin.

### 3.6. Ppdef1 Kills T. rubrum Growing on Keratin as a Sole Nutrient Source

**Figure 6:**
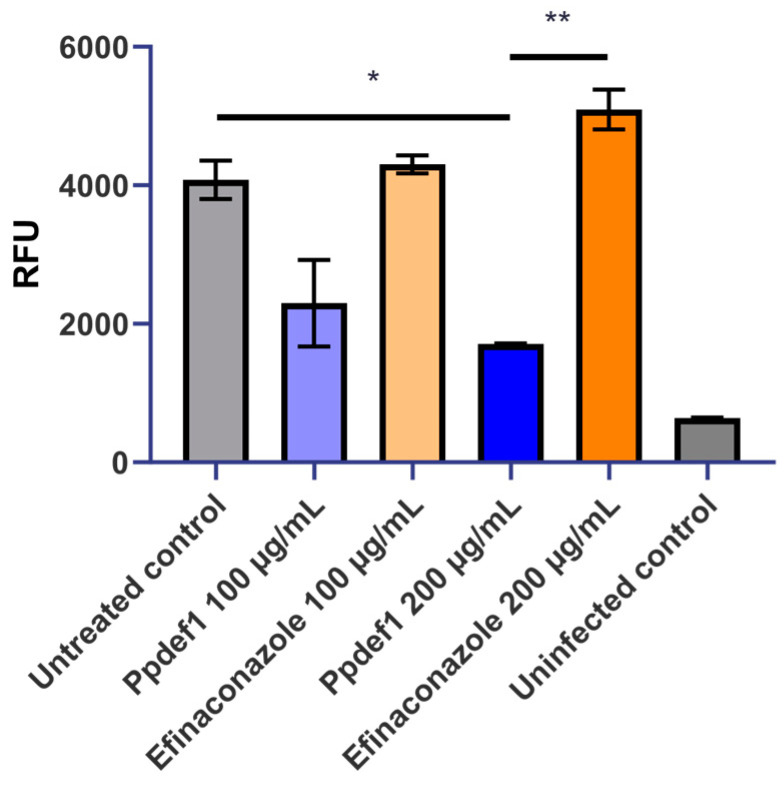
Ppdef1 kills *T. rubrum* growing on keratin as a sole nutrient source. Cell viability of hyphae grown on nail fragments for 72 h was monitored using PrestoBlue^®^. *T. rubrum* was treated with two concentrations of Ppdef1 (blue), efinaconazole (orange), or water (grey) for 24 h before cell viability was measured. Uninfected nails were included as a control (dark grey). Ppdef1 treatment at 200 µg/mL caused a significant decrease in the number of viable fungi compared to the untreated control (* *p* < 0.05) and 200 µg/mL efinaconazole (** *p* < 0.01). Efinaconazole did not cause a significant decrease in viable fungi at either concentration. Error bars indicate the standard error of the mean.

To confirm that Ppdef1 was able to kill fungal cells using keratin as a sole nutrient source, Ppdef1 was tested for its ability to kill *T. rubrum* growing on sterilized human nail fragments. The viability of fungal cells was assessed using PrestoBlue^®^ in the presence and absence of Ppdef1 and efinaconazole. Ppdef1 reduced the amount of viable *T. rubrum* growing on keratin as a sole nutrient source in a concentration-dependent manner for Ppdef1. At 100 or 200 µg/mL, Ppdef1 killed 51% and 66% of the hyphal biomass, respectively, relative to the untreated control. In comparison, efinaconazole had little impact on the amount of living biomass, even at 200 µg/mL (Figure 6). 

### 3.7. Ppdef1 Penetrates Human Nail Fragments

**Figure 7:**
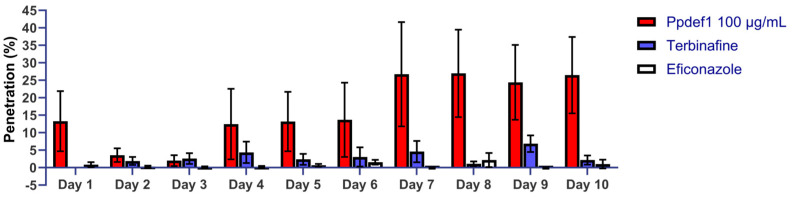
Ppdef1 penetrates human nails. Ppdef1 (1%), terbinafine (1%), and efinaconazole (10%) were applied to human fingernails daily for 10 days, and the amount of drug that had passed through the nail was monitored. The percent of the applied dose detected each day is indicated by the bars corresponding to each day. Greater than 20% of the Ppdef1 penetrated the nail on Days 7–10. In contrast, less than 5% of both terbinafine and efinaconazole penetrated the nails on all days, except for terbinafine on Days 7 and 9, where penetration was still less than 10%. Data are the average of six replicates for Ppdef1, and four replicates for terbinafine and efinaconazole. Error bars indicate the standard error of the mean.

To assess the ability of Ppdef1 to penetrate human nail, Ppdef1 drug product was applied daily to the surface of human nail fragments, and its passage through the nail was monitored daily for 10 days. An average of 13.3% of the applied dose penetrated the nails on the first day. This increased to approximately 26% penetration by Day 7 (Figure 7). A decrease in penetration was observed on Days 2 and 3 to 3.2% and 2.0%, respectively, but it increased to an average of 13.1% per day over Days 4, 5, and 6 with the average penetration per day at Days 7–10 of 26.1%. In contrast, the maximum level of terbinafine penetration on any one day was 7.0%, with only approximately 3.0% penetration observed on most days (Figure 7). The average penetration per day at Days 7–10 was only 3.8%. Efinaconazole had a maximum penetration of 2.2% on day 8 (Figure 7) and an average penetration per day of approximately 0.5% on Days 1–7, 9, and 10. The nail penetration data demonstrates that Ppdef1 has superior nail penetration data than either terbinafine or efinaconazole. 

### 3.8. Infected Nail Model

**Figure 8:**
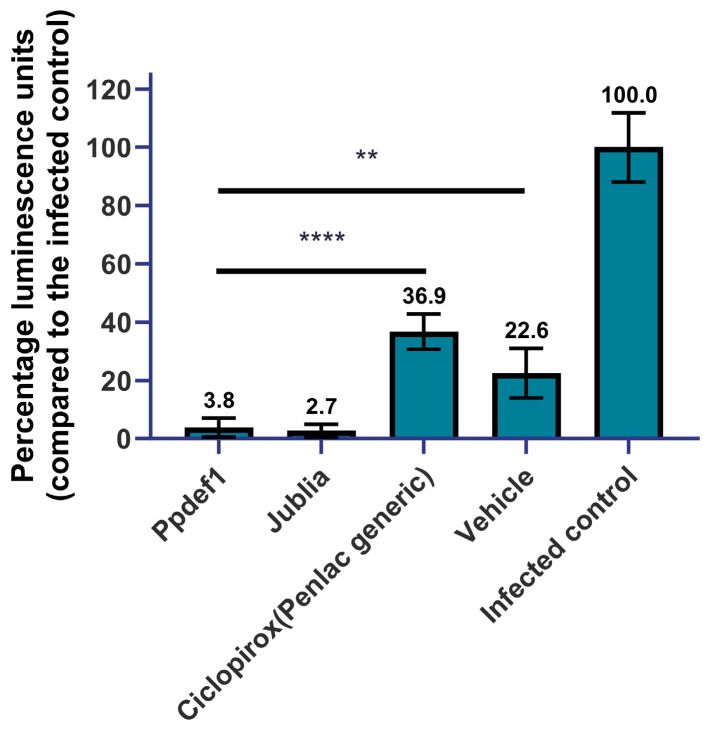
Ppdef1 is active in an infected nail model. *T. rubrum*-infected nails were treated with Ppdef1 reduced drug product, Jublia^®^, Penlac^®^, and Ppdef1 reduced base formulation (i.e., vehicle) for 7 days. At the end of the study, the amount of viable fungus remaining was assessed by measuring the amount of ATP recovered (presented as percentage luminescence units relative to the infected control). All three of the antifungal drugs as well as the Ppdef1 vehicle caused a significant decrease in viable fungi in the infected nail model with Ppdef1 and Jublia^®^ both resulting in less than 4 percent of the untreated infected control. Ppdef1 and Jublia^®^ caused a significantly greater fungal kill than ciclopirox (**** *p* < 0.0001) and vehicle (** *p* < 0.01). Error bars represent the standard error of the mean (*n* = 6, with the exception of ciclopirox, *n* = 3).

To confirm that Ppdef1 penetrates human nails and kills fungal pathogens, the penetration of Ppdef1 was tested in an infected nail model. The effect of the treatment on cell viability was assessed by measuring ATP levels in the hyphae at the end of the assay. A seven-day daily treatment with Ppdef1 reduced ATP levels by 96.2% relative to the untreated control (Figure 8), indicating that Ppdef1 had penetrated the human nails and eliminated the underlying fungi. The formulation in the absence of Ppdef1 (vehicle) reduced ATP levels by 77.4% relative to the uninfected control. Under the same conditions, Jublia^®^, containing the active ingredient efinaconazole (10%), reduced ATP levels by 97.3% relative to the untreated control. A generic version of the drug Penlac^®^, with the active ingredient ciclopirox (8%), only reduced viability by 63.1%. 

## 4. Discussion

Ppdef1 is a novel antifungal peptide with excellent activity against the pathogens that cause onychomycosis. It is a unique member of the extensive plant defensin family [18] from the plant *Picramnia pentandra*, an evergreen shrub that grows in parts of South America, the Caribbean, and South East North America. Ppdef1 is likely to have a different mechanism of antifungal activity because it shares less than 50% sequence identity with characterised defensins, and this is reduced to ~16% when the highly conserved cysteine and glycine residues that are essential for the defensin fold are not included. Another striking feature of Ppdef1 is an extended loop 5 region (Figure 1) that is very different in terms of sequence to all characterized plant defensins. Loop 5 is important for antifungal activity because it mediates the interaction with specific lipids and passage through the plasma membrane into the fungal cytoplasm [32,33,34,35]. Despite the low sequence conservation, the structure of Ppdef1 is very similar to that reported for other plant defensins apart from an extension of the β2- and β3- strands and the hairpin loop that joins them (loop 5, Figure 3A). The surface of Ppdef1 is predominantly positively charged apart from a small neutral patch on one side of the protruding loop 5 (Figure 3B). 

Ppdef1 has a potent antifungal activity on a range of human fungal pathogens, including *C. albicans* and *T. rubrum*, the most common causative agent of onychomycosis. For all pathogens tested, the MFC for Ppdef1 was only slightly higher than the MIC, which is consistent with a fungicidal rather than a fungistatic mode of action. This was confirmed using the viability stain propidium iodide (PI). Ppdef1 disrupted the membrane of *C. albicans* cells, leading to PI uptake in a concentration-dependent manner within just 30 min of treatment (Figure 4). At a concentration of 50 µg/mL, Ppdef1 killed over 60% of the cells within 30 min. In contrast, there was no significant cell death after treatment with an equivalent concentration of the antifungal drugs efinaconazole, terbinafine, or tavaborole over the same period, indicating that Ppdef1 kills cells more efficiently than these antifungal agents in vitro. The antifungal drug ciclopirox caused only a small amount of cell death (~20%) during this time, substantially less than Ppdef1. 

Efinaconazole is a member of the azole family of antimycotics and functions by preventing ergosterol biosynthesis by inhibiting sterol 14α-demethylase [36]. Azoles have a largely fungistatic mode of action, except at very high doses or against specific fungal species [37]. In addition, fungi can rapidly develop resistance to azole drugs through simple structural modifications of the enzyme target, the up-regulation of drug efflux pumps, or cellular stress responses [38]. Terbinafine is an allylamine that also inhibits ergosterol biosynthesis by inhibiting squalene epoxidase, a different enzyme in the biosynthesis pathway [39]. The associated build-up of squalene can be fungicidal. However, in this study, terbinafine did not kill fungal cells as rapidly as Ppdef1. Tavaborole is the first member of a novel class of antifungal drugs that functions by forming a stable adduct in the editing site of leucyl-tRNA synthetase, thereby blocking protein synthesis [40]. Tavaborole is fungistatic rather than fungicidal. Ciclopirox is also fungistatic but can be fungicidal at concentrations ~30-fold higher than the MIC [41]. The concentrations of ciclopirox used in this study were high relative to the MIC, explaining why some fungicidal activity was observed. However, despite the high concentration used, the fungicidal effects of ciclopirox were much lower than those obtained with Ppdef1. 

The rapid fungicidal activity of Ppdef1 will be beneficial for the treatment of onychomycosis because it will limit the re-establishment of the original infection after treatment has ceased. It is expected to also reduce the development of resistance because dead cells cannot mutate to become resistant to the antifungal agent. Defensins are thought to act on multiple cellular targets to exert their mechanism of action [12,42,43], further decreasing the likelihood of resistance developing. A pilot study comparing the development of resistance to the plant defensin NaD1 and caspofungin confirmed this hypothesis [44]. Well-known hot spot mutations in *FKS1* allowed the fungus to develop resistance to caspofungin more rapidly than to NaD1, where tolerance increased incrementally as mutations in various stress response genes occurred. Defensin tolerance was also associated with a fitness penalty, further decreasing the probability of resistance developing in the clinic.

During infection, dermatophytes break down keratin and use the products as a nutrient source [45]. As such, the growth of fungi in nails differs substantially, and is considerably slower, than the growth in a nutrient-rich medium. Consequently, current antifungal therapies are generally less effective against slower growing fungi because these fungi do not need to rapidly produce membrane and cell wall components. As such, fungi that grow either in the presence of keratin or that use keratin as a sole nutrient source can differ in their response to antifungal agents [46]. The effect of keratin on the activity of Ppdef1 was tested using *T. rubrum* that was growing in ½ PDB containing either hydrolysed animal keratin (from skin) or ground human nail. In both instances, *T. rubrum* grew more quickly in the presence of keratin, probably because of the increase in available nutrients. The presence of hydrolysed animal keratin did not affect the MIC of Ppdef1. Interestingly, the addition of ground nail material to the culture medium lowered the MIC of Ppdef1 by ~4-fold. Since the growth of *T. rubrum* was similar in the media containing ground nail material or hydrolysed keratin, it is unlikely that the ground nail material was contributing compounds that inhibited fungal growth. Plant defensins are known to act in synergy with other peptides that do not have any antifungal activity on their own [47,48,49]. Ground nail material may contain peptides that act in synergy with Ppdef1 to inhibit fungal growth. Human beta defensin 2 (hBD2) has been identified in human nail fragments, suggesting that the nail plate does contain innate immunity peptides [50].

To determine whether Ppdef1 could kill fungi growing on keratin as a sole nutrient source, *T. rubrum* was grown on nail fragments in the absence of any added nutrients before treatment with Ppdef1. Ppdef1 reduced the amount of living *T. rubrum* in a concentration-dependent manner, while efinaconazole had little impact on the amount of living biomass even at the highest concentrations tested (200 µg/mL). Fungicidal activity in the presence of keratin is hypothesised to correlate with the clinical efficacy for the topical onychomycosis products, ciclopirox and efinaconazole [46]. The complete cure rate for ciclopirox is reported at 5.5–8.5% versus 15.2–17.8% for efinaconazole [4,5]. 

To be effective as a treatment for onychomycosis, a topically applied product needs to pass through the nail plate to reach fungal hyphae growing in the nail bed. Despite its relatively large size of 5479 Da relative to efinaconazole (348 Da) and tavaborole (152 Da), Ppdef1 efficiently penetrated the nail plate. This occurred within 24 h and had a higher rate of penetration than either efinaconazole or terbinafine. The solubility of each drug in their respective vehicles was not assessed in this study and may affect the relative nail penetration. In contrast to other antifungal drugs used in topical onychomycosis therapies, Ppdef1 is highly positive charged and is hydrophilic, which likely enhances its passage through the hydrophilic keratin of the nail. Following the daily application of 10 µL of a 1% solution of Ppdef1 for 10 days, more than 150 µg had passed through the nail. Given the MIC for Ppdef1 is 13–30 µg/mL, this amount would likely be sufficient to exert fungicidal activity in the nail bed. The superior nail penetration of Ppdef1 compared to terbinafine and efinaconazole is consistent with the notion that the nail behaves like a hydrated gel and allows hydrophilic molecules to penetrate but prevents hydrophobic molecules from entering. This property of the nail has been demonstrated across multiple studies that compare nail penetration and hydrophobicity, or octanol water partition coefficients, where the nail penetration correlates with solubility in water [7,8,9].

Ppdef1 was effective in an infected nail model. It passed through the nail plate, retained antifungal activity, and killed *T. rubrum* growing on the underside of the nail. Ppdef1 reduced the number of viable *T. rubrum* hyphae (as measured by the amount of ATP) to the same level as the current best-in-class topical onychomycosis product, Jublia^®^. Interestingly, the vehicle for Ppdef1 also had some activity in the infected nail model. This vehicle has a slightly acidic pH that may retard the growth of the fungus once it penetrates, which would likely be ineffective in the buffered environment of the nail bed.

The rapid and efficient penetration of human nails by Ppdef1 combined with its rapid fungicidal activity, even in the presence of keratin, make it an excellent candidate for the topical treatment of onychomycosis.

## Figures and Tables

**Table 1:** MICs and MFCs for Ppdef1 against fungi that cause onychomycosis.

Fungal Pathogen	MIC (µg/mL)	MFC (µg/mL)
*Trichophyton rubrum*	20 ± 5	25 ± 5
*Trichophyton mentagrophytes*	13.3 ± 3.3	15 ± 5
*Candida albicans*	30 ± 0	30 ± 0
*Fusarium oxysporum*	15.8 ± 2.2	21.7 ± 1.7

## Data Availability

Data are available on request due to privacy restrictions. The data presented in this study are available on request from the corresponding author. The data are not publicly available due to company policy.

## Supplementary Materials

The following supporting information can be downloaded at: https://www.mdpi.com/article/10.3390/jof9111111/s1, Table S1: Statistical analysis of Ppdef1 structures.
