# Supplementary material for: The Plant Defensin Ppdef1 Is a Novel Topical Treatment for Onychomycosis
Source: J Fungi (Basel). 2023 Nov 17;9(11):1111. doi: 10.3390/jof9111111 (PMC10672221; doi:10.3390/jof9111111)
Supplement: Supplementary file 1 [file jof-09-01111-s001.zip › jof-2691802-supplementary.pdf]

Table S1. Statistical analysis of pezadeftide structures. All statistics are given as mean ± SD.

**Experimental restraints**	
Total no. distance restraints	419
Intraresidue	128
Sequential	132
Medium range, *i-j*<5	53
Long range, *i-j*≥5	106
Hydrogen bond restraints	24
Dihedral angle restraints	
Phi	38
psi	25
chi1	19
**Deviations from idealized geometry**	
Bond lengths (Å)	0.011 ± 0.000
Bond angles (deg)	1.076 ± 0.041
Impropers (deg)	1.36 ± 0.11
NOE (Å)	0.010 ± 0.002
cDih (deg)	0.056 ± 0.053
**Mean energies (kcal/mol)**	
Overall	−1626 ± 35
Bonds	20.2 ± 1.4
Angles	58.4 ± 5.3
Improper	23.3 ± 3.4
van Der Waals	−216.2 ± 7.2
NOE	0.04 ± 0.01
cDih	0.06 ±0.09
Electrostatic	−1738 ± 35
**Violations**	
NOE violations exceeding 0.2 Å	0
Dihedral violations exceeding 2.0 Å	0
**Rms deviation from mean structure, Å**	
Backbone atoms	1.59 ± 0.44
All heavy atoms	2.35 ± 0.45
Backbone atoms, residues 18-26,5-7,32-37,43-48	0.61 ± 0.14
All heavy atoms, residues 18-26,5-7,32-37,43-48	1.30 ± 0.23
**Stereochemical quality** (according to MolProbity)	
Residues in most favoured Ramachandran region, %	94.7 ± 2.2
Ramachandran outliers, %	0.6 ± 1.3
Unfavourable sidechain rotamers, %	0.3 ± 0.7
Clashscore, all atoms	14.6 ± 4.1
Overall MolProbity score	2.1 ± 0.1
